# The Gut Microbiota in the Pathogenesis and Therapeutics of Inflammatory Bowel Disease

**DOI:** 10.3389/fmicb.2018.02247

**Published:** 2018-09-25

**Authors:** Tao Zuo, Siew C. Ng

**Affiliations:** ^1^Department of Medicine and Therapeutics, Institute of Digestive Disease, LKS Institute of Health Science, The Chinese University of Hong Kong, Hong Kong, China; ^2^Faculty of Medicine, Center for Gut Microbiota Research, The Chinese University of Hong Kong, Hong Kong, China

**Keywords:** gut microbiota, bacteria, virobiota, mycobiota, helminths, diet, inflammatory bowel disease, fecal microbiota transplantation

## Abstract

In the twenty first century, the changing epidemiology of inflammatory bowel disease (IBD) globally with increasing disease incidence across many countries relates to the altered gut microbiota, due to a combinatorial effect of environmental factors, human immune responses and genetics. IBD is a gastrointestinal disease associated with a gut microbial dysbiosis, including an expansion of facultative anaerobic bacteria of the family Enterobacteriaceae. Advances in high-throughput sequencing enable us to entangle the gut microbiota in human health and IBD beyond the gut bacterial microbiota, expanding insights into the mycobiota, virobiota and helminthes. *Caudovirales* (viruses) and *Basidiomycota, Ascomycota*, and *Candida albicans* (fungi) are revealed to be increased in IBD. The deconvolution of the gut microbiota in IBD lays the basis for unveiling the roles of these various gut microbiota components in IBD pathogenesis and being conductive to instructing on future IBD diagnosis and therapeutics. Here we comprehensively elucidate the alterations in the gut microbiota in IBD, discuss the effect of diets in the gut microbiota in relation to IBD, and illustrate the potential of manipulation of gut microbiota for IBD therapeutics. The therapeutic strategy of antibiotics, prebiotics, probiotics and fecal microbiota transplantation will benefit the effective application of precision microbiome manipulation in IBD.

## Introduction

**Figure 1:**
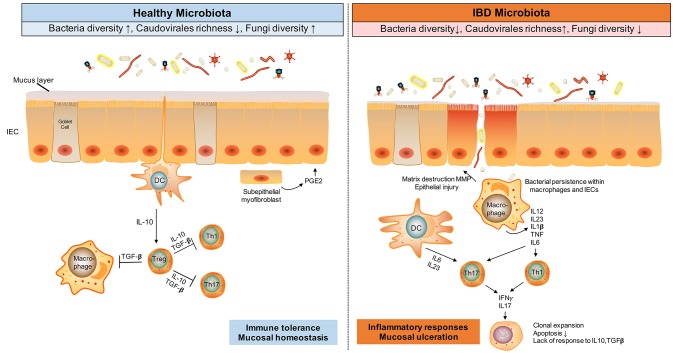
Gut microbiota alteration and immune responses in IBD. The gut microbes, including bacteria, viruses and fungi, and dysfunctional immune responses, engaging Tregs, T-helper 1 (Th1), and Th17, are implicated in IBD pathogenesis. During homestasis, gut microbes induce an immune tolerance phenotype in the host, whilst in inflammatory conditions like IBD, antigens from dysbiotic microbes activate Th1 and Th17 cells, resulting in tissue injury, decreased mucus layer, and microbial penetration and persistence in the intestinal tissues. This mucosal injury results in further uptake of microbial antigens, TLR ligands, and viable organisms that perpetuate the immune responses. TGF, transforming growth factor; TGF, transforming growth factor; MMP, matrix metalloproteinase; DC, dendritic cell.

Inflammatory bowel diseases (IBD), including Crohn's disease (CD) and ulcerative disease (UC), are proposed to result from an inappropriate immune response to the gut microbes in a genetically susceptible host. It is a chronic inflammatory disorder of the intestinal tract of an unknown cause. The incidence of IBD has increased in the western world since the midst of the twentieth century (Molodecky et al., 2012; Rocchi et al., 2012; Hammer et al., 2016). At the turn of the twenty-first century, it plateaued in some developed nations with a prevalence of up to 0.5% of the general population, while it is continuing to rise in developing nations (Benchimol et al., 2009, 2014; Kaplan, 2015). Etiological studies on IBD have centered on several factors, including host genetics and immune responses, the gut microbiota, and the importance of environmental stimuli in disease pathogenesis (Figure 1). Gut dysbiosis has been consistently shown to be associated with IBD. Due to the expansion in application of high-throughput deep sequencing technology in the past decade, we are able to gradually unveiling the role of the microbiome in development of IBD. These findings have improved our knowledge on the functional mechanisms of the microbiome in the pathogenesis of IBD.

The gut microbiota, comprising bacteria, fungi, viruses, and other microorganisms, operate as a secondary organ system with critical functions to the host. IBD is amongst the most extensively studied of all inflammatory diseases that are closely associated with the gut microbiome. In this review, we discuss the role of the gut microbiota, including the bacterial microbiota, mycobiota and virobiota in the development of IBD as well as microbiota-based therapeutic approaches in the treatment of IBD.

## Gut microbiome and inflammatory bowel disease (IBD)

### Bacterial microbiota

Bacterial microbiota is the most well-studied component of the gut microbiota, which inhabit its host in variable concentrations. In the gastrointestinal (GI) tract, it reaches an upper level in the colon of 10^11^ or 10^12^ cells/g of luminal contents (Dave et al., 2012). It was estimated that our gut contains ~1,000 bacterial species and 100-fold more genes than that found in the human genome (Ley et al., 2006; Qin et al., 2010). This bacterial community performs a diversity of important functions in the host, including educating the immune system (Round and Mazmanian, 2009), secreting enzymes for digesting substrates inaccessible to the host (El Kaoutari et al., 2013), and repressing detrimental microorganisms (O'Hara and Shanahan, 2006). Overall, the phyla *Firmicutes, Bacteroidetes, Actinobacteria* and *Verrucomicrobia* are the predominant constituents in the healthy gut microbiota (Jandhyala et al., 2015). The gut bacterial microbiome exhibit differences with regard to both the mucosal-to-luminal and proximal-to-distal gradients, displaying substantial variations between individuals (Turnbaugh et al., 2009; Qin et al., 2010; Bäckhed et al., 2012). The gut bacterial microbiota develops from a low diverse community at birth into a highly complex community with the introduction of diets by 9–12 months of age (Koenig et al., 2011; Backhed et al., 2015). The microbiota becomes stable and resilient to environmental perturbations, such as dietary changes or short-term antibiotic exposure (Dethlefsen and Relman, 2011; Wu et al., 2011). A multitude of factors have been shown to intervene with the gut microbiome, including age, genetics, diet and drugs (Yatsunenko et al., 2012; Maier et al., 2018; Zuo et al., 2018).

Gut microbes were demonstrated to be an essential factor in intestinal inflammation in IBD (Tamboli et al., 2004; Sartor, 2008). Some studies suggest that dysbiosis occur in IBD (Frank et al., 2007; Casen et al., 2015; Putignani et al., 2016; Halfvarson et al., 2017), and a broad microbial alteration pattern was revealed including reduction in diversity, decreased abundances of bacterial taxa within the Phyla *Firmicutes* and *Bacteroides*, and increases in the *Gammaproteobacteria* (Table 1; Frank et al., 2011; Morgan et al., 2012). In IBD, it has been consistently shown that there is a decrease in biodiversity, knowingly α diversity, and in species richness, a measure of the total number of species in a community. Patients with CD exhibited a reduced α diversity in the fecal microbiome, compared with healthy controls (Manichanh et al., 2006), and similar findings were found in monozygotic twins discordant for CD (Dicksved et al., 2008). This decreased diversity was partly linked to the temporal instability of the dominant taxa in IBD (Martinez et al., 2008). There is also reduced diversity in inflamed vs. non-inflamed tissues even within the same patient, and a lower bacterial load was observed at the inflamed regions in CD patients (Sepehri et al., 2007). A multicenter study that they investigated >1,000 treatment-naïve pediatric CD samples collected from multiple concurrent gastrointestinal locations (Gevers et al., 2014). They found that changes in bacteria, including increased *Veillonellaceae, Pasteurellacaea, Enterobacteriaceae*, and *Fusobacteriaceae*, and decreased *Bacteroidales, Erysipelotrichales*, and *Clostridiale*s, strongly correlated with disease status. This study also showed that rectal mucosa-associated microbiome profiling offered a feasible biomarker for the diagnosis of CD at the early stage of disease.

**Table 1:** Altered gut microbiota in IBD compared to healthy individuals in humans.

	**Decreased in IBD**	**Increased in IBD**
Microbial composition	*Bifidobacterium* sp.	*Proteobacteria*
	*Groups IV and XIVA Clostridium*	*Escherichia coli*, adherent/invasivea
	*Faecalibacterium prausnitzii*	*Fusobacterium* species
	*Roseburia* species	*Ruminococcus gnavusa*
	*Suterella* species	*Pasteurellaceae*
	*Bacteroides*	*Veillonellaceae*
	*Saccharomyces cerevisiae*	*Caudovirales Clavispora lusitaniae Kluyveromyces marxianus Candida albicans Candida tropicalis Cyberlindnera jadinii*
Microbial function	SCFAs, butyrate Butanoate and propanoate metabolism Amino acid biosynthesis	Auxotrophy Amino acid transport Sulfate transport Oxidative stress Type II secretion system Secretion of toxins

Human studies have shown that abundance of specific bacteria taxa were altered in IBD (Table 1). *Enterobacteriacae* bacteria are augmented both in patients with IBD and in mice (Lupp et al., 2007). *Escherichia coli*, particularly adherent-invasive *E. coli* (AIEC) strains, were isolated from ileal CD biopsies (Darfeuille-Michaud et al., 2004) and were also found in UC patients (Sokol et al., 2006). Meanwhile, mucosal samples showed more pronounced enrichment than fecal samples (Chassaing and Darfeuille-Michaud, 2011). It indicates that the inflammatory environment in IBD may favor the growth of this bacterial clade, *Enterobacteriaceae*. Anti-inflammatory drug, mesalamine, could attenuate intestinal inflammation and decrease the abundance of *Escherichia*/*Shigella* in IBD (Benjamin et al., 2012; Morgan et al., 2012).

*Fusobacteria* is another clade of adherent and invasive bacteria. Bacteria of the genus *Fusobacterium* principally colonize both the oral cavity and the gut. *Fusobacterium* species were present at a higher abundance in the colonic mucosa of patients with UC compared to healthy controls (Ohkusa et al., 2002, 2009). When administered by rectal enema in mice, *Fusobacterium varium* was able to cause colonic mucosal inflammation (Ohkusa et al., 2003). The invasive ability of human *Fusobacterium* bacteria correlate positively with host IBD severity (Strauss et al., 2011), indicating that invasive *Fusobacterium* species may impact IBD pathology. Beyond that, *Fusobacterium* species were documented to be abundantly present in tumor than in adjacent normal tissue in colorectal cancer (Castellarin et al., 2012; Kostic et al., 2012; Yu et al., 2017). Besides, human *Fusobacterium* isolates were reported to have a tumorigenesis role in mice (Kostic et al., 2013).

There are also specific groups of gut bacteria that may play a protective role against IBD. A range of bacterial species, most notably the genera *Lactobacillus, Bifidobacterium*, and *Faecalibacterium*, have been shown to be protective of the host from mucosal inflammation via several mechanisms, including the stimulation of the anti-inflammatory cytokine (Sokol et al., 2008), including IL-10 and down-regulation of inflammatory cytokines (Llopis et al., 2009). *Faecalibacterium prausnitzii*, has been shown to have anti-inflammatory properties and was undererrepresented in IBD (Sokol et al., 2009). The abundance of *F. prausnitzii* is significantly decreased while the abundance of *E. coli* is increased in ileal biopsies of CD specimens (Willing et al., 2009). CD patients with low abundances of *F. prausnitzii* in the mucosa are more likely have relapse after surgery (Sokol et al., 2008). In contrast, restoration of *F. prausnitzii* after recurrence is associated with maintenance of clinical remission of UC (Varela et al., 2013). Epidemiological data in humans suggested a protective effect of *Helicobacter pylori* (*H. pylori*) infection against the development of autoimmune diseases, including IBD (Papamichael et al., 2014; Rokkas et al., 2015). Laboratory data demonstrated that *H. pylori* could induce immune tolerance and limit inflammatory responses (Arnold et al., 2011; Oertli and Müller, 2012; Oertli et al., 2012).

In chemically-induced mice with colitis, the colitis phenotype is more severe in germ-free mice than in conventionally reared mice (Kitajima et al., 2001). The commensal microbes protect the host via colonization resistance, where commensals occupy niches within the host and prevent invasion by pathogens (Callaway et al., 2008), outcompete pathogenic bacteria (Kamada et al., 2012). Commensals have also showed functional effects on pathogens, such as dampening virulence-related gene expression (Medellin-Pena et al., 2007). In addition, the gut microbiota can modulate the host mucosal immune response. *Clostridium* and *Bacteroides* species could induce the expansion of regulatory T cells (Treg) and to mitigate intestinal inflammation (Atarashi et al., 2013). Other gut bacterial members can alleviate mucosal inflammation by regulating NF-κB activation (Kelly et al., 2004).

Some members of the gut microbiota have the ability to ferment dietary fiber resulting in the production of short-chain fatty acids (SCFAs), including acetate, propionate, and butyrate. SCFAs have been shown to exert multiple beneficial effects on mammalian energy metabolism. They are the primary energy source for colonic epithelial cells (Ahmad et al., 2000) and can induce the expansion of colonic Treg cells (Atarashi et al., 2013; Smith et al., 2013). The butyrate-producing genus *Faecalibacterium*, which belonged to *Ruminococcaceae*, is decreased in IBD, especially in ileal CD (Sokol et al., 2008, 2009; Xenoulis et al., 2008; Kang et al., 2010; Morgan et al., 2012). Other SCFA-producing bacteria, *Phascolarctobacterium* and *Roseburia* are also reduced in CD, whilst *Leuconostocaceae* is depleted in UC (Morgan et al., 2012). The gut microbiota co-evolves with the polysaccharide-rich diet, to synergistically enable the host efficiently extract energy from dietary fiber and protect them from inflammation (Willing et al., 2009). Lack of dietary fiber intake has been associated with the development of IBD (Galvez et al., 2005; Chiba et al., 2015; Pituch-Zdanowska et al., 2015). In addition, low fiber diet is associated with a low concentration of SCFAs (Galvez et al., 2005; Maslowski and Mackay, 2011).

### Fungal microbiota (mycobiota)

Fungi represent only a minor constituent of the gut microbiota and account for <0.1% of the total microbes (Qin et al., 2010). This may be an underestimation due to our current challenge to annotate fungi as a result of an incomplete fungal genomic database (Underhill and Iliev, 2014). Nevertheless, target region sequencing of marker genes, such as internal transcribed spacer (ITS) and 18S rRNA, mapping to annotated databases has contributed to an improved understanding of the mycobiota in the gut (Qin et al., 2010).

Fungi vary in composition at different body sites in humans (Underhill and Iliev, 2014). The GI tract, urogenital tract and oral cavity have the largest proportion of the taxa of the *Candida* genus which comprises up to 160 species (Soll et al., 1991; Huffnagle and Noverr, 2013). Species-specific colonization patterns of *Candida* are observed in mammals. *Candida albicans, C. parapsilosis* and *C. blabrata* are common in humans whereas *C. tropicalis* predominates in mice (Underhill and Iliev, 2014). In contrary to the relative stability of the bacterial microbiota over time, the mouse gut mycobiota have been observed to vary over time among mouse reared in cages suggesting an environmental influence on the gut fungi (Dollive et al., 2013). Competition between gut bacteria and fungi was observed. In humans and mice, a relatively long-term use of antibiotics can promote fungal overgrowth and infection (Noverr et al., 2004; Dollive et al., 2013). In line with these findings, antibiotic-induced fungal overgrowth in the gut predispose the host to the development of allergic airway in response to mold spore exposures (Noverr et al., 2004). These observations support a role for the gut mycobiota in the development of immune-mediated diseases.

In healthy individuals, *Saccharomyces, Candida*, and *Cladosporium* were the most predominate genera (Hoffmann et al., 2013). *Basidiomycota, Ascomycota*, and *C. albicans* have been shown to be significantly elevated in IBD patients (Ott et al., 2008; Hoffmann et al., 2013; Sokol et al., 2017). However, until recently the fungi composition in the mucosa is beginning to be elucidated. Liguori et al. evaluated the gut fungal composition in patients with CD and healthy individuals (Liguori et al., 2016). *Saccharomycetes* and *Tremellomycetes* classes, which belong to the phyla *Ascomycota* and *Basidiomycota*, respectively, are the most abundant taxa in the mucosal samples from healthy individuals. At a lower taxonomic level, these two classes can be divided into the genera *Candida, Debaryomyces, Saccharomyces, Malassezia, Sporobolomyces, Trichosporon, Wallemia*, together with a smaller proportion of unidentified *Filobasidiaceae* and *Xylariales*, In samples from CD subjects, the genus *Dioszegia* and species *Candida glabrata* predominate especially during a disease flare whereas *Trichosporon* and *Leptosphaeria* genera are decreased (Mckenzie et al., 1990; Standaert-Vitse et al., 2009; Schwiertz et al., 2010; Hansen et al., 2013; Mukhopadhya et al., 2015; Liguori et al., 2016). In addition, *Filobasidium uniguttulatum* and *Saccharomyces cerevisiae* were significantly elevated in non-inflamed mucosa, whereas *Xylariales* were increased in inflamed mucosa of CD (Liguori et al., 2016). In contrast with CD patients, UC patients showed a relatively decreased diversity of fungi. This might be due to an impairment of the inhibition of antimicrobial peptides against bacteria in the small bowel and altered bile acid reabsorption which can benefit the growth of fungi in patients with CD and not in UC. Overall these preliminary data suggest that an increased fungal load of *Candida* species and altered bacteria diversity may be associated with the pathogenic feature of CD.

There is also emerging evidence favoring a role for the gut mycobiota in IBD pathogenesis. A number of studies have investigated host immune responses against fungal cell wall derived molecules (Levitz, 2010). The glycoprotein cell wall components of the fungi, chitin, β-glucans and mannans can trigger the innate immune response, through receptors, such as dectin-1 (a C-type lectin receptor), Toll-like receptors (TLR2 and TLR4), components of the complement system, and members of the scavenger receptor family (CD5, SCARF1, and CD36). Activation of these molecules leads to downstream immune cascades engaging molecules, such as CARD9, IL-17, IL-22, ITAM, NFAT, and NF-κB (Sartor and Wu, 2017). Colitis in mouse can be exacerbated due to infiltration of fungi through disrupted mucosal barrier. Brun et al. and Iliev et al. suggested that fungi can invade the host by breaking down the mucosal barrier (Brun et al., 2007; Underhill and Iliev, 2014). Intestinal epithelial cells (IECs) act as a physical barrier to prevent foreign pathogens from invading the host. However, in IBD in humans and mice, prolonged inflammation along with the disruption of tight junction (TJ) Occludin and ZO-1, leads to loss of integrity of IECs. Pathogens like fungi and bacteria can therefore penetrate the mucosal barrier and activate TLRs, Dectin-1 and CARD9 in the lamina propria resulting into a more severe inflammatory phenotype (Brun et al., 2007; Iliev et al., 2012).

### Viral microbiota (virobiota)

With the thriving of high-throughput sequencing technologies (Virgin, 2014), the importance of the gut virobiota is being better appreciated. The virobiota (which is referred to as the viral component of the microbiota) comprises both eukaryotic viruses and prokaryotic bacteriophages. They contain more diverse biological entities than the gut bacterial microbiota (Lecuit and Eloit, 2013; Ogilvie and Jones, 2015). The gut virome in healthy humans is characterized predominantly by the bacteriophages temperate dsDNA *Caudovirales* and ssDNA *Microviridae* that latently infect their bacterial hosts but can generate progenies that may infect and kill other bacteria when under stress (Reyes et al., 2010; Minot et al., 2012, 2013; Waller et al., 2014). In health, gut bacteriophages show intensive variation between subjects while temporally are stable in each individual (Reyes et al., 2010; Minot et al., 2013).

Bacteriophages are posited to play a role in IBD, though their function in disease pathogenesis remains unequivocal (Perez-Brocal et al., 2013; Norman et al., 2015). Patients with CD exhibited a lower diversity but higher variability of the gut virome relative to controls (Perez-Brocal et al., 2013). The virome richness was increased in CD and UC patients from the United Kingdom, Chicago and Boston (Norman et al., 2015). Children with CD have more bacteriophages from the order *Caudovirales* in their tissues and intestinal washings compared to non-inflammatory controls (Wagner et al., 2013). Furthermore, *Caudovirales* virions have been obtained from washings of CD biopsies observed under electron microscopy (Lepage et al., 2008).

The expansion in bacteriophages in IBD could arise from commensal microbes entering lytic cycles or from new viruses introduction from the surrounding environment. Meanwhile, alteration in bacteriophage composition might have further impact on the bacterial microbiota ecology. Bacteriophages are shown to be primary drivers of bacterial fitness and diversity (Brüssow et al., 2004). Moreover, the gut microbiome is prone to be affected by bacteriophage invasion, resulting in changes in the bacterial abundance of specific species (Reyes et al., 2013). In the GI tract, bacteriophages engage in the horizontal transfer of genetic elements between bacterial populations, including those for antibiotic resistance and disease pathogenesis (Maiques et al., 2006; Zhang and LeJeune, 2008; Reyes et al., 2013). Hence, increased virome richness with widespread bacteriophage acquisition in IBD, potentially from external sources, could effectively change bacterial fitness. In mice, western diet could induce an expansion of *Caudovirales* (Kim and Bae, 2016), implicating a role for diet in altering the gut virome.

One potential role of enteric bacteriophages in IBD might be their direct interaction with the mammalian host. Bacteriophages can translocate from the GI lumen to systemic sites in murines (Górski et al., 2006), patients with CD and healthy controls (Parent and Wilson, 1971). They can also induce humoral immune responses (Uhr et al., 1962). Altogether, bacteriophages may act as immune ligands or antigens that boost host immunity and inflammation. On the other hand, some viruses, such as norovirus, can functionally replace the beneficial effect of commensal bacteria, ameliorating intestinal abnormalities in germ-free mice and diminishing susceptibility to intestinal damage caused by chemical injury and bacterial infection (Kernbauer et al., 2014). Beyond that, viruses attached to the mucosa could protect the epithelium against bacteria invasion, through binding interactions between Ig-Iike proteins exposed on the phage capsid and mucin glycoproteins on the mucosal surface (Barr J. et al., 2013; Barr J. J. et al., 2013).

There is also compelling evidence from mice showing that gut viruses play a causal role in chronic GI inflammation (Cadwell et al., 2010). Mice with a mutation in the gene ATG16L1 (an autophagy gene), which in humans predisposes to CD, are developed without symptom. However, gut norovirus infection led to the manifestation of the disease, although solely harboring the susceptible allele or having the virus alone did not produce the disease, suggesting the combinatorial effect of susceptibility gene and virus in disease pathogenesis and/or progression. In a chemically induced colitis mouse model, the beneficial effect of the gut virome on the gut mucosal immune homeostasis was demonstrated (Yang et al., 2016). Mice, administered a cocktail of anti-viral drugs followed by dextran sulfate sodium (DSS), had more severe colitis than animals treated with DSS alone, along with greater reduction in colon length and weight loss. The observed protective effects of the gut viruses were mediated synergistically by TLR3 and TLR7, but not individually. In favor of this, patients carrying mutations in both TLR3 and TLR7 have been shown to have higher rates of hospitalization when compared with IBD patients without mutations (Yang et al., 2016).

### Helminths

Gut helminthes are also considered to be one important gut microbial component coexisting in the gut with gut bacteria, fungi and viruses. The hygiene hypothesis suggests that a lack of early childhood exposure to symbiotic microorganisms and helminthic parasites increases susceptibility to immune mediated diseases in later life. Autoimmune and other immunologic diseases, such as IBD are highly prevalent in developed countries. However, with urbanization and environmental changes toward a more hygienic status, the uprising incidence of IBD appears to conincide with a diminished prevalence of helminth colonization in the host (Weinstock and Elliott, 2009).

Helminths are worm-like parasites, some of which have the potential to inhabit the GI tract and other regions of the body. Improvement in living standards in industrialized countries brings about environmental changes, which disrupt helminthic life cycles and lead to de-worming in humans. Before the twentieth century, every individual was likely to have at least one helminthic infection, mostly during early childhood, but this universal exposure has become rare in the twenty-first century (Elliott and Weinstock, 2012).

Helminths play an important immunoregulatory role in the host, and a lack of their presence has been associated with IBD development (Weinstock et al., 2002; Weinstock and Elliott, 2009; Ramanan et al., 2016). It was demonstrated by Ramanan et al. that helminths *Trichuris muris* and *Heligmosoides polygyrus* protected NOD2-deficient mice against intestinal inflammation, through inhibiting inflammatory *Bacteroides* species. This protective effect could be attributed to the functional influence of certain helminths on host physiology, exemplified by *Trichinella spiralis* (Motomura et al., 2009; Yang et al., 2014) and *Schistosoma mansoni* (Ruyssers et al., 2009) modulating Treg expansion, *Trichuris trichiura* (Broadhurst et al., 2010) upregulating Th22 response, and *Heligmosomoides polygyrus* (Elliott et al., 2008; Hang et al., 2013) down-regulating Th17 responses while expanding Treg population.

Compared with helminth-negative individuals, helminth-colonized subjects displayed higher gut bacterial diversity, in an indigenous Malaysian cohort (Lee et al., 2014). Orang Asli of the Temuan subtribe are helminthes-positive and they predominantly formed a cluster driven by the gut bacteria *Faecalibacterium* and *Prevotella*, compared with subjects in Kuala Lumpur (Ramanan et al., 2016). Those residing in the city in Kuala Lumpur clustered into another group characterized by *Bacteroides* (Ramanan et al., 2016). The concurrent disparity in helminth prevalence and microbiome configuration between rural and urban dwellers favors a linkage between helminth presence and bacterial microbiome structure, implicating a potential protective role for helminths in rural dwellers against IBD microbiota.

Helminth infections could cause increases in mucus and water secretion into the gut lumen, as measures for anti-inflammatory responses in the host (Stepek et al., 2002; Harnett and Harnett, 2006). In clinical trials of IBD, *Trichiuris suis* or pig whip worm were shown to be efficacious in patients with CD and UC (Summers et al., 2005a,b; Velthuis et al., 2010; Helmby, 2015).

## Diet and the gut microbiome

Diet is among the most important factors influencing the gut microbiota (Albenberg and Wu, 2014). Western diet is shown to predispose individuals to diseases including IBD, diabetes, obesity, hypercholesterolemia, and cardiovascular disease (Ley et al., 2008; Turnbaugh et al., 2008; Conlon and Bird, 2015; Agus et al., 2016; Carrillo-Larco et al., 2016). However, what or how certain diets protect against IBD is unclear, considering that urbanization is a significant risk factor for IBD (Soon et al., 2012). Study has shown that long-term diet can influence the composition and function of the gut microbiota (Muegge et al., 2011; Wu et al., 2011). Similarly, short-term diet can rapidly and reproducibly alter the gut microbiota (David et al., 2014). Compared to westerners on a western diet, inhabitants consuming a rural agrarian diet in rural areas and hunter–gatherers times showed a substantial higher diversity of the gut microbiota (De Filippo et al., 2010; Yatsunenko et al., 2012; Schnorr et al., 2014; Martínez et al., 2015; Obregon-Tito et al., 2015).

A western diet is associated with a decreased ratio of *Bacteroides* to *Firmicutes* and enhanced susceptibility to an increased presence of Adherent-Invasive *E. coli* (AIEC) infection (Agus et al., 2016). A recent review assessing the relationship between global IBD incidence and regional diets showed significant correlations between increased incidence of CD and increased consumptions of animal products, beer, honey, animal fats, and ghee, typical constituents of western diets. In contrast, an increased incidence of UC correlated with an increased consumption of pineapples and coffee products (Shivashankar et al., 2017). How these dietary products influence the gut microbiota and predispose individuals to IBD remains to be determined. Diet low in fiber was shown to be associated with a depletion of the microbial ecosystem in mice; this microbial extinction became irreversible and aggressive in the offspring over generations (Agus et al., 2016). The taxa driven to low abundances due to low-level intake of dietary microbiota-accessible carbohydrates were inefficiently transferred to the progeny (El Kaoutari et al., 2013). Mice transplanted with microbiota from humans on a typical unrestricted American diet (AMER) responded incompletely to plant-rich, calorie-restricted diet with optimized nutrient intake (CRON), while those transplanted with microbiota from CRON-consuming individuals responded strongly to both CRON and AMER diets (Griffin et al., 2017). These data imply that western diet may foster an irreversible microbial dysbiosis. In addition, a low-fiber diet was shown to exacerbate colitis and the expansion and activity of colonic mucus-degrading bacteria (Desai et al., 2016). Neither purified prebiotic fibers nor time-to-time diet oscillations between fiber-rich and fiber-deprived diet alleviate mucus layer degradation (Desai et al., 2016).

Specific types of diet are associated with alteration in the *Prevotella* to *Bacteroides* ratio. For instance, high consumption of amino acids, cholesterol, lipids and dairy products were shown to increase *Bacteroides* (Wu et al., 2011), while increase in *Prevotella* is boosted by consumption of sugar and other complex carbohydrates (De Filippo et al., 2010; Wu et al., 2011; Schnorr et al., 2014). The increased *Alistipes* and *Bilophila* were also linked to an animal-based diet (Wu et al., 2011; David et al., 2014). Vegans from non-westernized societies displayed an under-representation of *Bifidobacterium* (Wu et al., 2011), which might be ascribed to the absence of dairy products on their diet (Schnorr et al., 2014).

Diet has also been shown to impact on the gut mycobiota. In humans, increased *Candida* abundance is associated with diet high in carbohydrates, but not with diets high in protein, fatty acids and amino acids (Hoffmann et al., 2013). Consistently, in controlled feeding human study, *Candida* species in fecal samples were reduced in subjects consuming an animal-based diet and increased in subjects on a plant-based diet (David et al., 2014). The effects of diet on gut virobiota were also shown in humans and mice. In humans, dietary intervention was associated with a change in the virome to a new profile, in which individuals on the same diet converged (Minot et al., 2011). In mice, “western diet” could significantly enrich temperate bacteriophage *Caudovriales* both in the mucosa and luminal content (Kim and Bae, 2016). Interestingly, the community alteration of the virobiota occurred to a greater extent in the mucosa than in the lumen. Overall, diet has an impact on the gut mycobiota and virobiota. However, data and mechanistic study are still lacking, especially the associations among diet, mycobiota, virobiota and IBD.

Collectively, emerging animal and epidemiological studies have highlighted the necessity of preserving a diversified microbiome via diet.

## Manipulation of the microbiota in IBD therapeutics

### Antibiotics, probiotics and prebiotics

Antibiotics, probiotics and prebiotics have been utilized to treat IBD with varying results. Antibiotics have a modest effect in CD but data for probiotics and prebiotics are generally disappointing (Sartor and Wu, 2017). Single antibiotics could ameliorate Crohn's colitis and septic complications, and prevent post-resection recurrence, but has not been shown to be effective in patients with UC. Combinations of antibiotics might improve outcomes (Ohkusa et al., 2010; Turner et al., 2014) but the long-term use of antibiotics may induce development of antibiotic resistance in gut microbes.

Traditional probiotics have demonstrated limited effect in treating UC, the probiotic combination VSL#3 however (a probiotic preparation of eight live freeze-dried bacterial species, including *Lactobacillus casei, L. delbrueckii subsp. Bulgaricus, L. acidophilus, L. plantarum, Bifidobacterium longum, B. infanti*s, *B. breve*, and *Streptococcus salivarius subsp. Thermophiles*; Bibiloni et al., 2005) and *E. coli* Nissle were shown to reduce active inflammation and sustain remission (Wehkamp et al., 2004; Schultz, 2008). Contrarily, they do not benefit patients with CD. *F. prausnitzii* may have a protective effect on the intestine by producing barrier-enhancing and immunosuppressive SCFAs, stimulating Tregs to produce IL-10 thereby inhibiting exaggerated immune responses in IBD. In multiple mouse models, *F. prausnitzii, Clostridia* strains, and *B. fragilis* could reduce the severity of colitis (Sokol et al., 2008; Round et al., 2011; Atarashi et al., 2013). These beneficial microbes and their metabolites should be explored as therapeutic agents in treatment of IBD. Although the idea of providing dietary substrates, such as oligosaccharides and fiber, as a prebiotic means to selectively increase the abundance of SCFA-producing commensals is tantalizing, results to date have been not satisfactory (Sartor and Wu, 2017). Alternatively, it is feasible to block AIEC epithelial adherence, invasion and translocation via use of antibodies to flagellin and of antagonists to glycopolymers or FimH (Yan et al., 2015; Chassaing et al., 2016). Blocking the protease activity of *E. faecalis* or protease receptor binding has been shown to inhibit mucosal permeability (Steck et al., 2011; Maharshak et al., 2015). Thus, selectively blocking the virulence products of pathogenic microbes or their activity may diminish the dysbiotic bacteria in the gut in IBD. Recently, it is shown that precision editing of the gut microbiota by tungstate ameliorates colitis in mice (Zhu et al., 2018). Tungstate treatment could prevent gut inflammation as well as the dysbiotic expansion of Enterobacteriaceae by selectively inhibitting molybdenum-cofactor-dependent microbial respiratory pathways, while causing minimal changes in the microbiota composition under homeostatic conditions (Zhu et al., 2018).

Microbial markers have been shown to help in predicting which subset of patients are likely to have a positive response to treatment or who may undergo an aggressive disease course. Studies have shown that prognostic biomarkers from microbial profiling are instrumental to personalized therapy. For instance, microbial structure data, combined with level of apolipoprotein A1, can predict steroid-free remission in children newly diagnosed with CD (Haberman et al., 2014). Risk for post-operative recurrence of CD is linked to preoperative ileal concentrations of *F. prausnitzii* (Sokol et al., 2008). Risks for pouchitis post-colectomy in patients with UC can be predicted by the bacterial configuration (Machiels et al., 2017). In addition, microbial signatures correlate with response to therapy (Shaw et al., 2016) and dysbiosis is associated with relapse in patients after cessation of infliximab (Rajca et al., 2014).

There are a multitude of modern lifestyle associated factors that potentially associate with alterations of the gut microbiota (Table 2, any of these exposures may occur early in life). Studies from the West have shown that exposure of infants to antibiotics increases their risk for developing IBD (Patwa et al., 2011; Brito and Alm, 2016). More compelling evidence has been derived from human migration studies where migrants showed increased risks for IBD after migrating from developing to developed countries adopting an urbanized lifestyle (Probert et al., 1992; Barreiro-de Acosta et al., 2011). In a Canadian population-based inception and birth cohort study, rural residence in the first 1–5 years of life was associated with a lower risk of IBD (Benchimol et al., 2017). Early life colonization of microbes plays an essential role in the host immune system development (Gensollen et al., 2016). Further comprehensive appreciation of the underlying mechanisms would provide insights into the role of gut microbes in childhood in IBD pathogenesis.

**Table 2:** Factors associated with modern lifestyle that is potentially associated with alterations of the gut microbiota.

Modern lifestyle	Often live in an urban setting, surrounded by concrete Increased urbanization and migration to urban areas Birth in a hospital; increasing rate of cesarean delivery Small family size Sanitation of living spaces: environment colonized by resistant microorganisms (including resistant bacteria, fungi, and acari) Antibiotic usage early in life Frequent body wash with hot water and soap Low rate of *Helicobacter pylori* colonization Decline in endemic parasitism Food conserved by refrigeration Consumption of processed foods and food additives Increased pollution in developing nations
Traditional lifestyle	Vaginal delivery at home Large family size, crowding Livingin a rural setting in contact with soil microorganisms Ancestral colonization of the living environment No antibiotics in infant life Limited access to hot water and soap High rate of *Helicobacter pylori* colonization Parasitic worms common Food conserved by microbial fermentation Consumption of natural foods

### Fecal microbiota transplantation

Fecal microbiota transplantation (FMT), a highly effective therapy for recurrent *Clostridium difficile* infection (CDI) (Kassam et al., 2013), has gained substantial interest as a novel treatment for inflammatory bowel disease (IBD). The success of FMT in treating CDI is ascribed to restoration of the gut microbial homeostasis in patients with dysbiosis (Khoruts and Sadowsky, 2016). This approach was later extended to studies of other diseases, such as IBD (Borody et al., 2003) and metabolic syndrome (Vrieze et al., 2013). The evidence for the efficacy of FMT in treating IBD is equivocal. A systematic analysis of 18 studies that included 122 patients with IBD found that around 36–45% of patients achieved clinical remission during follow-up (Colman and Rubin, 2014). Subgroup analyses demonstrated a pooled estimate of clinical remission rate of 22% for UC and 61% for CD (Colman and Rubin, 2014).

Two placebo-controlled trials of FMT on patients with UC have shown conflicting results with one study demonstrating the importance of the donor effect. It was documented that some patients with UC experienced fevers and increased levels of C-reactive protein post-FMT (Angelberger et al., 2013). Disease flares in patients with UC or CD after FMT were also observed in the treatment of CDI (De Leon et al., 2013; Kelly et al., 2014a,b).

A recent multicenter, double-blind, randomized, placebo-controlled trial showed that intensive-dosing, with multidonor FMT induces clinical remission and endoscopic improvement in active ulcerative colitis and is associated with distinct microbial changes related to the outcome (Paramsothy et al., 2017). The primary outcome was achieved in 11 (27%) of 41 patients allocated to FMT compared with 3 (8%) of 40 subjects who were assigned to placebo (risk ratio 3.6, *p* = 0.021). Importantly, the microbial diversity increased and persisted after FMT (Paramsothy et al., 2017). These data are consistent with a more recent study showing the low intensity pooled donor FMT is also effective in active UC (Costello et al., 2017).

It has been reported that 30–50% of the donor's bacterial microbiota persist in the recipient after FMT (Li et al., 2016). Two studies have shown bacteriophage transfer from donor to recipient (Chehoud et al., 2016; Zuo et al., 2017) and in a pilot study donor virome richness is associated with the efficacy of FMT (Zuo et al., 2017). When donor Caudovirales virome richness was higher than that of the recipient, the recipient was more likely to be cured after FMT treatment (Zuo et al., 2017). Patients with IBD have been shown to harbor significantly higher virome richness than healthy household controls (Norman et al., 2015), which may account for the higher failure rate of FMT in treating IBD than in treating CDI (De Leon et al., 2013; Colman and Rubin, 2014; Kelly et al., 2015). These data highlights the importance of donor selection, where inclusion of a donor with high virome richness or pooled multiple donors is preferred.

The long-term consequence of FMT in treating diseases remains unclear. In the future, FMT will be likely substituted by the use of defined microbial consortia. It was proven in animals that such an approach was feasible and effective for the treatment of IBD (Atarashi et al., 2013).

## Perspectives

Efforts to date have effectively characterized the diverse constituents of the human gut microbiota in health and IBD. Bacterial microbiota is the most studied gut microbiota component though the function and strain-level resolution studies are still lacking. Beyond that, the under-studied gut microbiota components, viruses and fungi, and their inter-kingdom interactions with gut bacteria may have high impact in human health and IBD. These studies are still in its infancy. In summary, understanding the complexity of this gut ecosystem will require thorough mechanistic studies involving sophisticated molecular microbiologic techniques and animal studies to better define the role of different gut microbes in IBD pathogenesis and disease evolution. Precise interpretation of the cause and consequence of these microbial alterations will also require integration with host genetic polymorphisms and gene expression to allow proper comprehension of microbial-host interaction. Although microbe-based therapeutics is appealing, effective application of probiotics, prebiotics, antibiotics and FMT will require a personalized approach to identify defined subsets of patients that will benefit most from such a strategy.

## Author contributions

TZ and SN devised the concept, acquired data, and wrote the manuscript.

### Conflict of interest statement

The authors declare that the research was conducted in the absence of any commercial or financial relationships that could be construed as a potential conflict of interest.

## References

[B1] Agus A. Denizot J. Thevenot J. Martinez-Medina M. Massier S. Sauvanet P.. (2016). Western diet induces a shift in microbiota composition enhancing susceptibility to Adherent-Invasive, *E. coli* infection and intestinal inflammation. Sci. Rep. 6:19032. 10.1038/srep19032 26742586 PMC4705701

[B2] Ahmad M. S. Krishnan S. Ramakrishna B. S. Mathan M. Pulimood A. B. Murthy S. N. (2000). Butyrate and glucose metabolism by colonocytes in experimental colitis in mice. Gut 46, 493–499. 10.1136/gut.46.4.493 10716678 PMC1727901

[B3] Albenberg L. G. Wu G. D. (2014). Diet and the intestinal microbiome: associations, functions, and implications for health and disease. Gastroenterology 146, 1564–1572. 10.1053/j.gastro.2014.01.058 24503132 PMC4216184

[B4] Angelberger S. Reinisch W. Makristathis A. Lichtenberger C. Dejaco C. Papay P.. (2013). Temporal bacterial community dynamics vary among ulcerative colitis patients after fecal microbiota transplantation. Am. J. Gastroenterol. 108, 1620–1630. 10.1038/ajg.2013.257 24060759

[B5] Arnold I. C. Lee J. Y. Amieva M. R. Roers A. Flavell R. A. Sparwasser T.. (2011). Tolerance rather than immunity protects from *Helicobacter* pylori–induced gastric preneoplasia. Gastroenterology 140, 199–209. 10.1053/j.gastro.2010.06.047 20600031 PMC3380634

[B6] Atarashi K. Tanoue T. Oshima K. Suda W. Nagano Y. Nishikawa H.. (2013). T-reg induction by a rationally selected mixture of Clostridia strains from the human microbiota. Nature 500, 232–236. 10.1038/nature12331 23842501

[B7] Bäckhed F. Fraser C. M. Ringel Y. Sanders M. E. Sartor R. B. Sherman P. M.. (2012). Defining a healthy human gut microbiome: current concepts, future directions, and clinical applications. Cell Host Microbe 12, 611–622. 10.1016/j.chom.2012.10.012 23159051

[B8] Backhed F. Roswall J. Peng Y. Feng Q. Jia H. Kovatcheva-Datchary P.. (2015). Dynamics and stabilization of the human gut microbiome during the first year of life. Cell Host Microbe 17:852. 10.1016/j.chom.2015.05.012 26308884

[B9] Barr J. Auro R. Furlan M. Whiteson K. Talago N. Paul L. (2013). Bacteriophage adhered to mucus provide a novel mucosal immune system. J. Immunol. 190:61.8.

[B10] Barr J. J. Auro R. Furlan M. Whiteson K. L. Erb M. L. Pogliano J.. (2013). Bacteriophage adhering to mucus provide a non-host-derived immunity. Proc. Natl. Acad. Sci. U.S.A. 110, 10771–10776. 10.1073/pnas.1305923110 23690590 PMC3696810

[B11] Barreiro-de Acosta M. Alvarez Castro A. Souto R. Iglesias M. Lorenzo A. Dominguez-Munoz J. E. (2011). Emigration to western industrialized countries: a risk factor for developing inflammatory bowel disease. J. Crohns Colitis 5, 566–569. 10.1016/j.crohns,.2011.05.009 22115376

[B12] Benchimol E. I. Guttmann A. Griffiths A. M. Rabeneck L. Mack D. R. Brill H.. (2009). Increasing incidence of paediatric inflammatory bowel disease in Ontario, Canada: evidence from health administrative data. Gut 58, 1490–1497. 10.1136/gut.2009.188383 19651626

[B13] Benchimol E. I. Kaplan G. G. Otley A. R. Nguyen G. C. Underwood F. E. Guttmann A.. (2017). Rural and urban residence during early life is associated with a lower risk of inflammatory bowel disease: a population-based inception and birth cohort study. Am. J. Gastroenterol. 112, 1412–1422. 10.1038/ajg.2017.208 28741616 PMC5596205

[B14] Benchimol E. I. Manuel D. G. Guttmann A. Nguyen G. C. Mojaverian N. Quach P.. (2014). Changing age demographics of inflammatory bowel disease in ontario, canada: a population-based cohort study of epidemiology trends. Inflamm. Bowel Dis. 20, 1761–1769. 10.1097/MIB.0000000000000103 25159453

[B15] Benjamin J. L. Hedin C. R. Koutsoumpas A. Ng S. C. McCarthy N. E. Prescott N. J.. (2012). Smokers with active Crohn's disease have a clinically relevant dysbiosis of the gastrointestinal microbiota. Inflamm. Bowel Dis. 18, 1092–1100. 10.1002/ibd.21864 22102318

[B16] Bibiloni R. Fedorak R. N. Tannock G. W. Madsen K. L. Gionchetti P. Campieri M.. (2005). VSL# 3 probiotic-mixture induces remission in patients with active ulcerative colitis. Am. J. Gastroenterol. 100, 1539–1546. 10.1111/j.1572-0241.2005.41794.x 15984978

[B17] Borody T. J. Warren E. F. Leis S. Surace R. Ashman O. (2003). Treatment of ulcerative colitis using fecal bacteriotherapy. J. Clin. Gastroenterol. 37, 42–47. 10.1097/00004836-200307000-00012 12811208

[B18] Brito I. L. Alm E. J. (2016). Tracking strains in the microbiome: insights from metagenomics and models. Front. Microbiol. 7:712. 10.3389/fmicb.2016.00712 27242733 PMC4871868

[B19] Broadhurst M. J. Leung J. M. Kashyap V. McCune J. M. Mahadevan U. McKerrow J. H.. (2010). IL-22(+) CD4(+) T Cells are associated with therapeutic trichuris trichiura infection in an ulcerative colitis patient. Sci. Transl. Med. 2:60ra88. 10.1126/scitranslmed.3001500 21123809

[B20] Brun P. Castagliuolo I. Di Leo V. Buda A. Pinzani M. Palù G.. (2007). Increased intestinal permeability in obese mice: new evidence in the pathogenesis of nonalcoholic steatohepatitis. Am. J. Physiol. Gastr. L 292, G518–G525. 10.1152/ajpgi.00024.2006 17023554

[B21] Brüssow H. Canchaya C. Hardt W. D. (2004). Phages and the evolution of bacterial pathogens: From genomic rearrangements to lysogenic conversion. Microbiol. Mol. Biol. R 68, 560–602. 10.1128/MMBR.68.3.560-602.2004 15353570 PMC515249

[B22] Cadwell K. Patel K. K. Maloney N. S. Liu T. C. Ng A. C. Storer C. E.. (2010). Virus-plus-susceptibility gene interaction determines Crohn's disease gene Atg16L1 phenotypes in intestine. Cell 141, 1135–1145. 10.1016/j.cell.2010.05.009 20602997 PMC2908380

[B23] Callaway T. R. Edrington T. S. Anderson R. C. Harvey R. B. Genovese K. J. Kennedy C. N.. (2008). Probiotics, prebiotics and competitive exclusion for prophylaxis against bacterial disease. Anim. Health Res. Rev. 9, 217–225. 10.1017/S1466252308001540 19102792

[B24] Carrillo-Larco R. M. Bernabé-Ortiz A. Pillay T. D. Gilman R. H. Sanchez J. F. Poterico J. A.. (2016). Obesity risk in rural, urban and rural-to-urban migrants: prospective results of the PERU MIGRANT study. Int. J. Obes. 40, 181–185. 10.1038/ijo.2015.140 26228458 PMC4677453

[B25] Casen C. Vebo H. C. Sekelja M. Hegge F. T. Karlsson M. K. Ciemniejewska E.. (2015). Deviations in human gut microbiota: a novel diagnostic test for determining dysbiosis in patients with IBS or IBD. Aliment. Pharmacol. Ther. 42, 71–83. 10.1111/apt.13236 25973666 PMC5029765

[B26] Castellarin M. Warren R. L. Freeman J. D. Dreolini L. Krzywinski M. Strauss J.. (2012). *Fusobacterium* nucleatum infection is prevalent in human colorectal carcinoma. Genome Res. 22, 299–306. 10.1101/gr.126516.111 22009989 PMC3266037

[B27] Chassaing B. Darfeuille-Michaud A. (2011). The commensal microbiota and enteropathogens in the pathogenesis of inflammatory bowel diseases. Gastroenterology 140, 1720–1728. 10.1053/j.gastro.2011.01.054 21530738

[B28] Chassaing B. Tran H. Gewirtz A. (2016). O-007 Colitis vaccine: flagellin-elicited immunity keeps motile bacteria in check and protects against intestinal inflammation. Inflamm. Bowel Dis. 22:S3 10.1097/01.MIB.0000480045.11503.c5

[B29] Chehoud C. Dryga A. Hwang Y. Nagy-Szakal D. Hollister E. B. Luna R. A.. (2016). Transfer of viral communities between human individuals during fecal microbiota transplantation. mBio 7:e00322. 10.1128/mBio.00322-16 27025251 PMC4817255

[B30] Chiba M. Tsuji T. Nakane K. Komatsu M. (2015). High amount of dietary fiber not harmful but favorable for Crohn disease. Perm. J. 19, 58–91. 10.7812/TPP/14-124 25663207 PMC4315379

[B31] Colman R. J. Rubin D. T. (2014). Fecal microbiota transplantation as therapy for inflammatory bowel disease: a systematic review and meta-analysis. J. Crohns Colitis 8, 1569–1581. 10.1016/j.crohns.2014.08.006 25223604 PMC4296742

[B32] Conlon M. A. Bird A. R. (2015). The impact of diet and lifestyle on gut microbiota and human health. Nutrients 7, 17–44. 10.3390/nu7010017 25545101 PMC4303825

[B33] Costello S. Waters O. Bryant R. Katsikeros R. Makanyanga J. Schoeman M. (2017). Short duration, low intensity pooled faecal microbiota transplantation induces remission in patients with mild-moderately active ulcerative colitis: a randomised controlled trial. J. Crohns Colitis 11, S23 10.1093/ecco-jcc/jjx002.035

[B34] Darfeuille-Michaud A. Boudeau J. Bulois P. Neut C. Glasser A. L. Barnich N.. (2004). High prevalence of adherent-invasive *Escherichia coli* associated with ileal mucosa in Crohn's disease. Gastroenterology 127, 412–421. 10.1053/j.gastro.2004.04.061 15300573

[B35] Dave M. Higgins P. D. Middha S. Rioux K. P. (2012). The human gut microbiome: current knowledge, challenges, and future directions. Transl. Res. 160, 246–257. 10.1016/j.trsl.2012.05.003 22683238

[B36] David L. A. Maurice C. F. Carmody R. N. Gootenberg D. B. Button J. E. Wolfe B. E.. (2014). Diet rapidly and reproducibly alters the human gut microbiome. Nature 505, 559–563. 10.1038/nature12820 24336217 PMC3957428

[B37] De Filippo C. Cavalieri D. Di Paola M. Ramazzotti M. Poullet J. B. Massart S.. (2010). Impact of diet in shaping gut microbiota revealed by a comparative study in children from Europe and rural Africa. Proc. Natl. Acad. Sci. U.S.A. 107, 14691–14696. 10.1073/pnas.1005963107 20679230 PMC2930426

[B38] De Leon L. M. Watson J. B. Kelly C. R. (2013). Transient flare of ulcerative colitis after fecal microbiota transplantation for recurrent clostridium difficile infection. Clin. Gastroenterol. Hepatol. 11, 1036–1038. 10.1016/j.cgh.2013.04.045 23669309

[B39] Desai M. S. Seekatz A. M. Koropatkin N. M. Kamada N. Hickey C. A. Wolter M.. (2016). A dietary fiber-deprived gut microbiota degrades the colonic mucus barrier and enhances pathogen susceptibility. Cell 167, 1339–1353.e21. 10.1016/j.cell.2016.10.043 27863247 PMC5131798

[B40] Dethlefsen L. Relman D. A. (2011). Incomplete recovery and individualized responses of the human distal gut microbiota to repeated antibiotic perturbation. Proc. Natl. Acad. Sci. U.S.A. 108, 4554–4561. 10.1073/pnas.1000087107 20847294 PMC3063582

[B41] Dicksved J. Halfvarson J. Rosenquist M. Järnerot G. Tysk C. Apajalahti J.. (2008). Molecular analysis of the gut microbiota of identical twins with Crohn's disease. Isme J. 2, 716–727. 10.1038/ismej.2008.37 18401439

[B42] Dollive S. Chen Y. Y. Grunberg S. Bittinger K. Hoffmann C. Vandivier L.. (2013). Fungi of the murine gut: episodic variation and proliferation during antibiotic treatment. PLoS ONE 8:71806. 10.1371/journal.pone.0071806 23977147 PMC3747063

[B43] El Kaoutari A. Armougom F. Gordon J. I. Raoult D. Henrissat B. (2013). The abundance and variety of carbohydrate-active enzymes in the human gut microbiota. Nat. Rev. Microbiol. 11, 497–504. 10.1038/nrmicro3050 23748339

[B44] Elliott D. E. Metwali A. Leung J. Setiawan T. Blum A. M. Ince M. N.. (2008). Colonization with *Heligmosomoides polygyrus* suppresses mucosal IL-17 production. J. Immunol. 181, 2414–2419. 10.4049/jimmunol.181.4.2414 18684931 PMC4242718

[B45] Elliott D. E. Weinstock J. V. (2012). Helminth-host immunological interactions: prevention and control of immune-mediated diseases. Ann. N. Y. Acad. Sci. 1247, 83–96. 10.1111/j.1749-6632.2011.06292.x 22239614 PMC3744090

[B46] Frank D. N. Robertson C. E. Hamm C. M. Kpadeh Z. Zhang T. Chen H.. (2011). Disease phenotype and genotype are associated with shifts in intestinal-associated microbiota in inflammatory bowel diseases. Inflamm. Bowel Dis. 17, 179–184. 10.1002/ibd.21339 20839241 PMC3834564

[B47] Frank D. N. St Amand A. L. Feldman R. A. Boedeker E. C. Harpaz N. Pace N. R. (2007). Molecular-phylogenetic characterization of microbial community imbalances in human inflammatory bowel diseases. Proc. Natl. Acad. Sci. U.S.A. 104, 13780–13785. 10.1073/pnas.0706625104 17699621 PMC1959459

[B48] Galvez J. Rodríguez-Cabezas M. E. Zarzuelo A. (2005). Effects of dietary fiber on inflammatory bowel disease. Mol. Nutr. Food Res. 49, 601–608. 10.1002/mnfr.200500013 15841496

[B49] Gensollen T. Iyer S. S. Kasper D. L. Blumberg R. S. (2016). How colonization by microbiota in early life shapes the immune system. Science 352, 539–544. 10.1126/science.aad9378 27126036 PMC5050524

[B50] Gevers D. Kugathasan S. Denson L. A. Vázquez-Baeza Y. Van Treuren W. Ren B.. (2014). The treatment-naive microbiome in new-onset Crohn's disease. Cell Host Microbe 15, 382–392. 10.1016/j.chom.2014.02.005 24629344 PMC4059512

[B51] Górski A. Wazna E. Dabrowska B. W. Dabrowska K. Switala-Jelen K. Miedzybrodzki R. (2006). Bacteriophage translocation. FEMS Immunol. Med. Mic. 46, 313–319. 10.1111/j.1574-695X.2006.00044.x 16553803

[B52] Griffin N. W. Ahern P. P. Cheng J. Heath A. C. Ilkayeva O. Newgard C. B.. (2017). Prior dietary practices and connections to a human gut microbial metacommunity alter responses to diet interventions. Cell Host Microbe 21, 84–96. 10.1016/j.chom.2016.12.006 28041931 PMC5234936

[B53] Haberman Y. Tickle T. L. Dexheimer P. J. Kim M. O. Tang D. Karns R.. (2014). Pediatric Crohn disease patients exhibit specific ileal transcriptome and microbiome signature. J. Clin. Invest. 124, 3617–3633. 10.1172/JCI75436 25003194 PMC4109533

[B54] Halfvarson J. Brislawn C. J. Lamendella R. Vázquez-Baeza Y. Walters W. A. Bramer L. M.. (2017). Dynamics of the human gut microbiome in inflammatory bowel disease. Nat. Microbiol. 2:17004 10.1038/nmicrobiol.2017.4 28191884 PMC5319707

[B55] Hammer T. Nielsen K. R. Munkholm P. Burisch J. Lynge E. (2016). The faroese IBD study: incidence of inflammatory bowel diseases across 54 years of population-based data. J. Crohns Colitis 10, 934–942. 10.1093/ecco-jcc/jjw050 26933031 PMC4962362

[B56] Hang L. Blum A. M. Setiawan T. Urban J. P. Stoyanoff K. M. Weinstock J. V. (2013). Heligmosomoides polygyrus bakeri infection activates colonic Foxp3(+) T cells enhancing their capacity to prevent colitis. J. Immunol. 191, 1927–1934. 10.4049/jimmunol.1201457 23851695 PMC3790665

[B57] Hansen R. Mukhopadhya I. Meharg C. Russell R. Berry S. El-Omar E. (2013). The role of the fungal microbiota in the pathogenesis of de-novo paediatric inflammatory bowel disease using next generation sequencing. Gut 62, A32 10.1136/gutjnl-2013-304907.073 PMC4392392 25522934

[B58] Harnett W. Harnett M. M. (2006). Filarial nematode secreted product ES-62 is an anti-inflammatory agent: therapeutic potential of small molecule derivatives and ES-62 peptide mimetics. Clin. Exp. Pharmacol. Physiol. 33, 511–518. 10.1111/j.1440-1681.2006.04400.x 16700887

[B59] Helmby H. (2015). Human helminth therapy to treat inflammatory disorders- where do we stand? BMC Immunol. 16:12. 10.1186/s12865-015-0074-3 25884706 PMC4374592

[B60] Hoffmann C. Dollive S. Grunberg S. Chen J. Li H. Z. Wu G. D.. (2013). Archaea and fungi of the human gut microbiome: correlations with diet and bacterial residents. PLoS ONE 8:e66019. 10.1371/journal.pone.0066019 23799070 PMC3684604

[B61] Huffnagle G. B. Noverr M. C. (2013). The emerging world of the fungal microbiome. Trends Microbiol. 21, 334–341. 10.1016/j.tim.2013.04.002 23685069 PMC3708484

[B62] Iliev I. D. Funari V. A. Taylor K. D. Nguyen Q. Reyes C. N. Strom S. P.. (2012). Interactions between commensal fungi and the C-type lectin receptor dectin-1 influence colitis. Science 336, 1314–1317. 10.1126/science.1221789 22674328 PMC3432565

[B63] Jandhyala S. M. Talukdar R. Subramanyam C. Vuyyuru H. Sasikala M. Nageshwar Reddy D. (2015). Role of the normal gut microbiota. World J. Gastroenterol. 21, 8787–8803. 10.3748/wjg.v21.i29.8787 26269668 PMC4528021

[B64] Kamada N. Chen G. Núñez G. (2012). A complex microworld in the gut: harnessing pathogen-commensal relations. Nat. Med. 18, 1190–1191. 10.1038/nm.2900 22869189

[B65] Kang S. Denman S. E. Morrison M. Yu Z. Dore J. Leclerc M.. (2010). Dysbiosis of fecal microbiota in Crohn's disease patients as revealed by a custom phylogenetic microarray. Inflamm. Bowel Dis. 16, 2034–2042. 10.1002/ibd.21319 20848492

[B66] Kaplan G. G. (2015). The global burden of IBD: from 2015 to 2025. Nat. Rev. Gastro. Hepat. 12, 720–727. 10.1038/nrgastro.2015.150 26323879

[B67] Kassam Z. Lee C. H. Yuan Y. Hunt R. H. (2013). Fecal microbiota transplantation for Clostridium difficile infection: systematic review and meta-analysis. Am. J. Gastroenterol. 108, 500–508. 10.1038/ajg.2013.59 23511459

[B68] Kelly C. R. Ihunnah C. Fischer M. Khoruts A. Surawicz C. Afzali A.. (2014b). Fecal microbiota transplant for treatment of clostridium difficile infection in immunocompromised patients. Am. J. Gastroenterol. 109, 1065–1071. 10.1038/ajg.2014.133 24890442 PMC5537742

[B69] Kelly C. R. Kahn S. A. Kashyap P. (2015). Update on fecal microbiota transplantation 2015: indications, methodologies, mechanisms, and outlook (vol 149, pg 223, 2015). Gastroenterology 149, 1644–1644. 10.1053/j.gastro.2015.05.008 25982290 PMC4755303

[B70] Kelly C. R. Ziud H. Kahn S. (2014a). New diagnosis of Crohn's colits 6 weeks after fecal microbiota transplantation. Inflamm. Bowel Dis. 20:S21 10.1097/01.MIB.0000456762.32133.2e

[B71] Kelly D. Campbell J. I. King T. P. Grant G. Jansson E. A. Coutts A. G.. (2004). Commensal anaerobic gut bacteria attenuate inflammation by regulating nuclear-cytoplasmic shuttling of PPAR-[gamma] and RelA. Nat. Immunol. 5, 104–112. 10.1038/ni1018 14691478

[B72] Kernbauer E. Ding Y. Cadwell K. (2014). An enteric virus can replace the beneficial function of commensal bacteria. Nature 516, 94–98. 10.1038/nature13960 25409145 PMC4257755

[B73] Khoruts A. Sadowsky M. J. (2016). Understanding the mechanisms of faecal microbiota transplantation. Nat. Rev. Gastro. Hepat. 13, 508–516. 10.1038/nrgastro.2016.98 27329806 PMC5909819

[B74] Kim M. S. Bae J. W. (2016). Spatial disturbances in altered mucosal and luminal gut viromes of diet-induced obese mice. Environ. Microbiol. 18, 1498–1510. 10.1111/1462-2920.13182 26690305

[B75] Kitajima S. Morimoto M. Sagara E. Shimizu C. Ikeda Y. (2001). Dextran sodium sulfate-induced colitis in germ-free IQI/Jic mice. Exp. Anim. Tokyo 50, 387–395. 10.1538/expanim.50.387 11769541

[B76] Koenig J. E. Spor A. Scalfone N. Fricker A. D. Stombaugh J. Knight R.. (2011). Succession of microbial consortia in the developing infant gut microbiome. Proc. Natl. Acad. Sci. U.S.A. 108, 4578–4585. 10.1073/pnas.1000081107 20668239 PMC3063592

[B77] Kostic A. D. Chun E. Robertson L. Glickman J. N. Gallini C. A. Michaud M.. (2013). *Fusobacterium* nucleatum potentiates intestinal tumorigenesis and modulates the tumor-immune microenvironment. Cell Host Microbe 14, 207–215. 10.1016/j.chom.2013.07.007 23954159 PMC3772512

[B78] Kostic A. D. Gevers D. Pedamallu C. S. Michaud M. Duke F. Earl A. M.. (2012). Genomic analysis identifies association of *Fusobacterium* with colorectal carcinoma. Genome Res. 22, 292–298. 10.1101/gr.126573.111 22009990 PMC3266036

[B79] Lecuit M. Eloit M. (2013). The human virome: new tools and concepts. Trends Microbiol. 21, 510–515. 10.1016/j.tim.2013.07.001 23906500 PMC7172527

[B80] Lee S. C. Tang M. S. Lim Y. A. Choy S. H. Kurtz Z. D. Cox L. M.. (2014). Helminth colonization is associated with increased diversity of the gut microbiota. PLoS Negl. Trop. Dis. 8:e2880. 10.1371/journal.pntd.0002880 24851867 PMC4031128

[B81] Lepage P. Colombet J. Marteau P. Sime-Ngando T. Doré J. Leclerc M. (2008). Dysbiosis in inflammatory bowel disease: a role for bacteriophages? Gut 57, 424–425. 10.1136/gut.2007.134668 18268057

[B82] Levitz S. M. (2010). Innate recognition of fungal cell walls. PLoS Pathog. 6:1000758. 10.1371/journal.ppat.1000758 20421940 PMC2858700

[B83] Ley R. E. Hamady M. Lozupone C. Turnbaugh P. J. Ramey R. R. Bircher J. S.. (2008). Evolution of mammals and their gut microbes. Science 320, 1647–1651. 10.1126/science.1155725 18497261 PMC2649005

[B84] Ley R. E. Peterson D. A. Gordon J. I. (2006). Ecological and evolutionary forces shaping microbial diversity in the human intestine. Cell 124, 837–848. 10.1016/j.cell.2006.02.017 16497592

[B85] Li S. S. Zhu A. Benes V. Costea P. I. Hercog R. Hildebrand F.. (2016). Durable coexistence of donor and recipient strains after fecal microbiota transplantation. Science 352, 586–589. 10.1126/science.aad8852 27126044

[B86] Liguori G. Lamas B. Richard M. L. Brandi G. da Costa G. Hoffmann T. W.. (2016). Fungal dysbiosis in mucosa-associated microbiota of Crohn's disease patients. J. Crohns Colitis 10, 296–305. 10.1093/ecco-jcc/jjv209 26574491 PMC4957473

[B87] Llopis M. Antolin M. Carol M. Borruel N. Casellas F. Martinez C.. (2009). *Lactobacillus* casei downregulates commensals' inflammatory signals in Crohn's disease mucosa. Inflamm. Bowel Dis. 15, 275–283. 10.1002/ibd.20736 18839424

[B88] Lupp C. Robertson M. L. Wickham M. E. Sekirov I. Champion O. L. Gaynor E. C.. (2007). Host-mediated inflammation disrupts the intestinal microbiota and promotes the overgrowth of Enterobacteriaceae. Cell Host Microbe 2, 119–129. 10.1016/j.chom.2007.06.010 18005726

[B89] Machiels K. Sabino J. Vandermosten L. Joossens M. Arijs I. de Bruyn M.. (2017). Specific members of the predominant gut microbiota predict pouchitis following colectomy and IPAA in UC. Gut 66, 79–88. 10.1136/gutjnl-2015-309398 26423113

[B90] Maharshak N. Huh E. Y. Paiboonrungruang C. Shanahan M. Thurlow L. Herzog J.. (2015). Enterococcus faecalis gelatinase mediates intestinal permeability via protease-activated receptor 2. Infect. Immun. 83, 2762–2770. 10.1128/IAI.00425-15 25916983 PMC4468563

[B91] Maier L. Pruteanu M. Kuhn M. Zeller G. Telzerow A. Anderson E. E.. (2018). Extensive impact of non-antibiotic drugs on human gut bacteria. Nature 555, 623–628. 10.1038/nature25979 29555994 PMC6108420

[B92] Maiques E. Ubeda C. Campoy S. Salvador N. Lasa I. Novick R. P.. (2006). Beta-lactam antibiotics induce the SOS response and horizontal transfer of virulence factors in Staphylococcus aureus. J. Bacteriol. 188, 2726–2729. 10.1128/JB.188.7.2726-2729.2006 16547063 PMC1428414

[B93] Manichanh C. Rigottier-Gois L. Bonnaud E. Gloux K. Pelletier E. Frangeul L.. (2006). Reduced diversity of faecal microbiota in Crohn's disease revealed by a metagenomic approach. Gut 55, 205–211. 10.1136/gut.2005.073817 16188921 PMC1856500

[B94] Martinez C. Antolin M. Santos J. Torrejon A. Casellas F. Borruel N.. (2008). Unstable composition of the fecal microbiota in ulcerative colitis during clinical remission. Am. J. Gastroenterol. 103, 643–648. 10.1111/j.1572-0241.2007.01592.x 18341488

[B95] Martínez I. Stegen J. C. Maldonado-Gomez M. X. Eren A. M. Siba P. M. Greenhill A. R.. (2015). The gut microbiota of rural papua new guineans: composition, diversity patterns, and ecological processes. Cell Rep. 11, 527–538. 10.1016/j.celrep.2015.03.049 25892234

[B96] Maslowski K. M. Mackay C. R. (2011). Diet, gut microbiota and immune responses. Nat. Immunol. 12, 5–9. 10.1038/ni0111-5 21169997

[B97] Mckenzie H. Main J. Pennington C. R. Parratt D. (1990). Antibody to selected strains of saccharomyces-cerevisiae (Bakers and Brewers-Yeast) and Candida-Albicans in Crohns-disease. Gut 31, 536–538. 10.1136/gut.31.5.536 2190866 PMC1378569

[B98] Medellin-Pena M. J. Wang H. F. Johnson R. Anand S. Griffiths M. W. (2007). Probiotics affect virulence-related gene expression in *Escherichia coli* O157: H7. Appl. Environ. Microb. 73, 4259–4267. 10.1128/AEM.00159-07 17496132 PMC1932779

[B99] Minot S. Bryson A. Chehoud C. Wu G. D. Lewis J. D. Bushman F. D. (2013). Rapid evolution of the human gut virome. Proc. Natl. Acad. Sci. U.S.A. 110, 12450–12455. 10.1073/pnas.1300833110 23836644 PMC3725073

[B100] Minot S. Grunberg S. Wu G. D. Lewis J. D. Bushman F. D. (2012). Hypervariable loci in the human gut virome. Proc. Natl. Acad. Sci. U.S.A. 109, 3962–3966. 10.1073/pnas.1119061109 22355105 PMC3309749

[B101] Minot S. Sinha R. Chen J. Li H. Z. Keilbaugh S. A. Wu G. D.. (2011). The human gut virome: inter-individual variation and dynamic response to diet. Genome Res. 21, 1616–1625. 10.1101/gr.122705.111 21880779 PMC3202279

[B102] Molodecky N. A. Soon I. S. Rabi D. M. Ghali W. A. Ferris M. Chernoff G.. (2012). Increasing incidence and prevalence of the inflammatory bowel diseases with time, based on systematic review. Gastroenterology 142, 46–54. 10.1053/j.gastro.2011.10.001 22001864

[B103] Morgan X. C. Tickle T. L. Sokol H. Gevers D. Devaney K. L. Ward D. V.. (2012). Dysfunction of the intestinal microbiome in inflammatory bowel disease and treatment. Genome Biol. 13:R79. 10.1186/gb-2012-13-9-r79 23013615 PMC3506950

[B104] Motomura Y. Wang H. Deng Y. El-Sharkawy R. T. Verdu E. F. Khan W. I. (2009). Helminth antigen-based strategy to ameliorate inflammation in an experimental model of colitis. Clin. Exp. Immunol. 155, 88–95. 10.1111/j.1365-2249.2008.03805.x 19016806 PMC2665684

[B105] Muegge B. D. Kuczynski J. Knights D. Clemente J. C. González A. Fontana L.. (2011). Diet drives convergence in gut microbiome functions across mammalian phylogeny and within humans. Science 332, 970–974. 10.1126/science.1198719 21596990 PMC3303602

[B106] Mukhopadhya I. Hansen R. Meharg C. Thomson J. M. Russell R. K. Berry S. H.. (2015). The fungal microbiota of de-novo paediatric inflammatory bowel disease. Microbes Infect. 17, 304–310. 10.1016/j.micinf.2014.12.001 25522934 PMC4392392

[B107] Norman J. M. Handley S. A. Baldridge M. T. Droit L. Liu C. Y. Keller B. C.. (2015). Disease-specific alterations in the enteric virome in inflammatory bowel disease. Cell 160, 447–460. 10.1016/j.cell.2015.01.002 25619688 PMC4312520

[B108] Noverr M. C. Noggle R. M. Toews G. B. Huffnagle G. B. (2004). Role of antibiotics and fungal microbiota in driving pulmonary allergic responses. Infect. Immun. 72, 4996–5003. 10.1128/IAI.72.9.4996-5003.2004 15321991 PMC517468

[B109] Obregon-Tito A. J. Tito R. Y. Metcalf J. Sankaranarayanan K. Clemente J. C. Ursell L. K.. (2015). Subsistence strategies in traditional societies distinguish gut microbiomes. Nat. Commun. 6:6505. 10.1038/ncomms7505 25807110 PMC4386023

[B110] Oertli M. Müller A. (2012). *Helicobacter pylori* targets dendritic cells to induce immune tolerance, promote persistence and confer protection against allergic asthma. Gut Microbes 3, 566–571. 10.4161/gmic.21750 22895083 PMC3495795

[B111] Oertli M. Sundquist M. Hitzler I. Engler D. B. Arnold I. C. Reuter S.. (2012). DC-derived IL-18 drives Treg differentiation, murine *Helicobacter* pylori–specific immune tolerance, and asthma protection. J. Clin. Invest. 122, 1082–1096. 10.1172/JCI61029 22307326 PMC3287234

[B112] Ogilvie L. A. Jones B. V. (2015). The human gut virome: a multifaceted majority. Front. Microbiol. 6:918. 10.3389/fmicb.2015.00918 26441861 PMC4566309

[B113] O'Hara A. M. Shanahan F. (2006). The gut flora as a forgotten organ. EMBO Rep. 7, 688–693. 10.1038/sj.embor.7400731 16819463 PMC1500832

[B114] Ohkusa T. Kato K. Terao S. Chiba T. Mabe K. Murakami K.. (2010). Newly developed antibiotic combination therapy for ulcerative colitis: a double-blind placebo-controlled multicenter trial. Am. J. Gastroenterol. 105, 1820–1829. 10.1038/ajg.2010.84 20216533

[B115] Ohkusa T. Okayasu I. Ogihara T. Morita K. Ogawa M. Sato N. (2003). Induction of experimental ulcerative colitis by *Fusobacterium varium* isolated from colonic mucosa of patients with ulcerative colitis. Gut 52, 79–83. 10.1136/gut.52.1.79 12477765 PMC1773498

[B116] Ohkusa T. Sato N. Ogihara T. Morita K. Ogawa M. Okayasu I. (2002). Fusobacterium varium localized in the colonic mucosa of patients with ulcerative colitis stimulates species-specific antibody. J. Gastroenterol. Hepatol. 17, 849–853. 10.1046/j.1440-1746.2002.02834.x 12164960

[B117] Ohkusa T. Yoshida T. Sato N. Watanabe S. Tajiri H. Okayasu I. (2009). Commensal bacteria can enter colonic epithelial cells and induce proinflammatory cytokine secretion: a possible pathogenic mechanism of ulcerative colitis. J. Med. Microbiol. 58, 535–545. 10.1099/jmm.0.005801-0 19369513 PMC2887547

[B118] Ott S. J. Kuhbacher T. Musfeldt M. Rosenstiel P. Hellmig S. Rehman A.. (2008). Fungi and inflammatory bowel diseases: alterations of composition and diversity. Scand. J. Gastroentero. 43, 831–841. 10.1080/00365520801935434 18584522

[B119] Papamichael K. Konstantopoulos P. Mantzaris G. J. (2014). *Helicobacter* pylori infection and inflammatory bowel disease: is there a link? World J. Gastroenterol. 20:6374. 10.3748/wjg.v20.i21.6374 24914359 PMC4047323

[B120] Paramsothy S. Kamm M. A. Kaakoush N. O. Walsh A. J. van den Bogaerde J. Samuel D.. (2017). Multidonor intensive faecal microbiota transplantation for active ulcerative colitis: a randomised placebo-controlled trial. Lancet 389, 1218–1228. 10.1016/S0140-6736(17)30182-4 28214091

[B121] Parent K. Wilson I. D. (1971). Mycobacteriophage in Crohn's disease. Gut 12, 1019–1020. 10.1136/gut.12.12.1019 5157132 PMC1411989

[B122] Patwa L. G. Fan T. J. Tchaptchet S. Liu Y. Lussier Y. A. Sartor R. B.. (2011). Chronic intestinal inflammation induces stress-response genes in commensal *Escherichia coli*. Gastroenterology 141, 1842–1851.e1-10. 10.1053/j.gastro,.2011.06.064 21726510 PMC3624969

[B123] Perez-Brocal V. Garcia-Lopez R. Vazquez-Castellanos J. F. Nos P. Beltran B. Latorre A.. (2013). Study of the viral and microbial communities associated with Crohn's disease: a metagenomic approach. Clin. Transl. Gastroen. 4:e36. 10.1038/ctg.2013.9 23760301 PMC3696940

[B124] Pituch-Zdanowska A. Banaszkiewicz A. Albrecht P. (2015). The role of dietary fibre in inflammatory bowel disease. Prz. Gastroenterol. 10, 135–141. 10.5114/pg.2015.52753 26516378 PMC4607699

[B125] Probert C. S. Jayanthi V. Pinder D. Wicks A. C. Mayberry J. F. (1992). Epidemiologic-study of ulcerative proctocolitis in indian migrants and the indigenous population of leicestershire. Gut 33, 687–693. 10.1136/gut.33.5.687 1307684 PMC1379303

[B126] Putignani L. Del Chierico F. Vernocchi P. Cicala M. Cucchiara S. Dallapiccola B.. (2016). Gut microbiota dysbiosis as risk and premorbid factors of IBD and IBS along the childhood-adulthood transition. Inflamm. Bowel Dis. 22, 487–504. 10.1097/MIB.0000000000000602 26588090

[B127] Qin J. Li R. Raes J. Arumugam M. Burgdorf K. S. Manichanh C.. (2010). A human gut microbial gene catalog established by metagenomic sequencing. Nature 464, 59–65. 10.1038/nature08821 20203603 PMC3779803

[B128] Rajca S. Grondin V. Louis E. Vernier-Massouille G. Grimaud J. C. Bouhnik Y.. (2014). Alterations in the intestinal microbiome (Dysbiosis) as a predictor of relapse after infliximab withdrawal in Crohn's disease. Inflamm. Bowel Dis. 20, 978–986. 10.1097/MIB.0000000000000036 24788220

[B129] Ramanan D. Bowcutt R. Lee S. C. Tang M. S. Kurtz Z. D. Ding Y.. (2016). Helminth infection promotes colonization resistance via type 2 immunity. Science 352, 608–612. 10.1126/science.aaf3229 27080105 PMC4905769

[B130] Reyes A. Haynes M. Hanson N. Angly F. E. Heath A. C. Rohwer F.. (2010). Viruses in the faecal microbiota of monozygotic twins and their mothers. Nature 466, 334–338. 10.1038/nature09199 20631792 PMC2919852

[B131] Reyes A. Wu M. McNulty N. P. Rohwer F. L. Gordon J. I. (2013). Gnotobiotic mouse model of phage-bacterial host dynamics in the human gut. Proc. Natl. Acad. Sci. U.S.A. 110, 20236–20241. 10.1073/pnas.1319470110 24259713 PMC3864308

[B132] Rocchi A. Benchimol E. I. Bernstein C. N. Bitton A. Feagan B. Panaccione R.. (2012). Inflammatory bowel disease: a Canadian burden of illness review. Can J. Gastroenterol. 26, 811–817. 10.1155/2012/984575 23166905 PMC3495699

[B133] Rokkas T. Gisbert J. P. Niv Y. O'morain C. (2015). The association between *Helicobacter pylori* infection and inflammatory bowel disease based on meta-analysis. United Eur Gastroenterol. J. 3, 539–550. 10.1177/2050640615580889 26668747 PMC4669512

[B134] Round J. L. Lee S. M. Li J. Tran G. Jabri B. Chatila T. A.. (2011). The toll-like receptor 2 pathway establishes colonization by a commensal of the human microbiota. Science 332, 974–977. 10.1126/science.1206095 21512004 PMC3164325

[B135] Round J. L. Mazmanian S. K. (2009). The gut microbiota shapes intestinal immune responses during health and disease. Nat. Rev. Immunol. 9, 313–323. 10.1038/nri2515 19343057 PMC4095778

[B136] Ruyssers N. E. De Winter B. Y. De Man J. G. Loukas A. Pearson M. S. Weinstock J. V.. (2009). Therapeutic potential of helminth soluble proteins in TNBS-induced colitis in mice. Inflamm. Bowel Dis. 15, 491–500. 10.1002/ibd.20787 19023900

[B137] Sartor R. B. (2008). Microbial influences in inflammatory bowel diseases. Gastroenterology 134, 577–594. 10.1053/j.gastro.2007.11.059 18242222

[B138] Sartor R. B. Wu G. D. (2017). Roles for intestinal bacteria, viruses, and fungi in pathogenesis of inflammatory bowel diseases and therapeutic approaches. Gastroenterology 152, 327–339. 10.1053/j.gastro.2016.10.012 27769810 PMC5511756

[B139] Schnorr S. L. Candela M. Rampelli S. Centanni M. Consolandi C. Basaglia G.. (2014). Gut microbiome of the Hadza hunter-gatherers. Nat. Commun. 5:3654 10.1038/ncomms4654 24736369 PMC3996546

[B140] Schultz M. (2008). Clinical use of *E. coli* Nissle 1917 in inflammatory bowel disease. Inflamm. Bowel Dis. 14, 1012–1018. 10.1002/ibd.20377 18240278

[B141] Schwiertz A. Jacobi M. Frick J. Markus R. Rusch K. Kohler H. (2010). Microbiota in pediatric inflammatory bowel disease. J. Pediatr. Gastroenterol. Nutri. 50, E105–E105. 10.1016/j.jpeds.2010.02.046 20400104

[B142] Sepehri S. Kotlowski R. Bernstein C. N. Krause D. O. (2007). Microbial diversity of inflamed and noninflamed gut biopsy tissues in inflammatory bowel disease. Inflamm. Bowel Dis. 13, 675–683. 10.1002/ibd.20101 17262808

[B143] Shaw K. A. Bertha M. Hofmekler T. Chopra P. Vatanen T. Srivatsa A.. (2016). Dysbiosis, inflammation, and response to treatment: a longitudinal study of pediatric subjects with newly diagnosed inflammatory bowel disease. Genome Med. 8:75. 10.1186/s13073-016-0331-y 27412252 PMC4944441

[B144] Shivashankar R. Beauvais J. C. Lewis J. D. (2017). The relationship of regional diets with global incidence rates of inflammatory bowel disease. Gastroenterology 152, S975–S976. 10.1016/S0016-5085(17)33306-1

[B145] Smith P. M. Howitt M. R. Panikov N. Michaud M. Gallini C. Bohlooly-y M.. (2013). The microbial metabolites, short-chain fatty acids, regulate colonic Treg cell homeostasis. Science 341, 569–573. 10.1126/science.1241165 23828891 PMC3807819

[B146] Sokol H. Leducq V. Aschard H. Pham H. P. Jegou S. Landman C.. (2017). Fungal microbiota dysbiosis in IBD. Gut 66, 1039–1048. 10.1136/gutjnl-2015-310746 26843508 PMC5532459

[B147] Sokol H. Lepage P. Seksik P. Dore J. Marteau P. (2006). Temperature gradient gel electrophoresis of fecal 16S rRNA reveals active *Escherichia coli* in the microbiota of patients with ulcerative colitis. J. Clin. Microbiol. 44, 3172–3177. 10.1128/JCM.02600-05 16954244 PMC1594675

[B148] Sokol H. Pigneur B. Watterlot L. Lakhdari O. Bermudez-Humaran L. G. Gratadoux J. J.. (2008). *Faecalibacterium* prausnitzii is an anti-inflammatory commensal bacterium identified by gut microbiota analysis of Crohn disease patients. Proc. Natl. Acad. Sci. U.S.A. 105, 16731–16736. 10.1073/pnas.0804812105 18936492 PMC2575488

[B149] Sokol H. Seksik P. Furet J. P. Firmesse O. Nion-Larmurier I. Beaugerie L.. (2009). Low counts of *Faecalibacterium* prausnitzii in colitis microbiota. Inflamm. Bowel Dis. 15, 1183–1189. 10.1002/ibd.20903 19235886

[B150] Soll D. R. Galask R. Schmid J. Hanna C. Mac K. Morrow B. (1991). Genetic dissimilarity of commensal strains of candida spp carried in different anatomical locations of the same healthy women. J. Clin. Microbiol. 29, 1702–1710. 1761692 10.1128/jcm.29.8.1702-1710.1991 PMC270187

[B151] Soon S. Molodecky N. A. Rabi D. M. Ghali W. A. Barkema H. W. Kaplan G. G. (2012). The relationship between urban environment and the inflammatory bowel diseases: a systematic review and meta-analysis. BMC Gastroenterol. 12:51. 10.1186/1471-230X-12-51 22624994 PMC3517531

[B152] Standaert-Vitse A. Sendid B. Joossens M. Francois N. Vandewalle-El Khoury P. Branche J.. (2009). Candida albicans Colonization and ASCA in familial Crohn's Disease. Am. J. Gastroenterol. 104, 1745–1753. 10.1038/ajg.2009.225 19471251

[B153] Steck N. Hoffmann M. Sava I. Kim S. Hahne H. Schemann M.. (2011). *Enterococcus faecalis* metalloprotease compromises epithelial barrier and contributes to intestinal inflammation. Int. J. Med. Microbiol. 301, 106–106. 10.1053/j.gastro.2011.05.035 21699778

[B154] Stepek G. Auchie M. Tate R. Watson K. Russell D. G. Devaney E.. (2002). Expression of the filarial nematode phosphorylcholine-containing glycoprotein, ES62, is stage specific. Parasitology 125, 155–164. 10.1017/S0031182002001920 12211608

[B155] Strauss J. Kaplan G. G. Beck P. L. Rioux K. Panaccione R. Devinney R.. (2011). Invasive potential of gut mucosa-derived *Fusobacterium* nucleatum positively correlates with IBD status of the host. Inflamm. Bowel Dis. 17, 1971–1978. 10.1002/ibd.21606 21830275

[B156] Summers R. W. Elliott D. E. Urban J. F. Thompson R. Weinstock J. V. (2005a). Trichuris suis therapy in Crohn's disease. Gut 54, 87–90. 10.1136/gut.2004.041749 15591509 PMC1774382

[B157] Summers R. W. Elliott D. E. Urban J. F. Thompson R. A. Weinstock J. V. (2005b). Trichuris suis therapy for active ulcerative colitis: a randomized controlled trial. Gastroenterology 128, 825–832. 10.1053/j.gastro.2005.01.005 15825065

[B158] Tamboli C. P. Neut C. Desreumaux P. Colombel J. F. (2004). Dysbiosis as a prerequisite for IBD. Gut 53, 1057. 10.1136/gut.53.1.1 15194668 PMC1774115

[B159] Turnbaugh P. J. Baeckhed F. Fulton L. Gordon J. I. (2008). Diet-induced obesity is linked to marked but reversible alterations in the mouse distal gut microbiome. Cell Host Microbe 3, 213–223. 10.1016/j.chom.2008.02.015 18407065 PMC3687783

[B160] Turnbaugh P. J. Hamady M. Yatsunenko T. Cantarel B. L. Duncan A. Ley R. E.. (2009). A core gut microbiome in obese and lean twins. Nature 457, 480–484. 10.1038/nature07540 19043404 PMC2677729

[B161] Turner D. Levine A. Kolho K. L. Shaoul R. Ledder O. (2014). Combination of oral antibiotics may be effective in severe pediatric ulcerative colitis: a preliminary report. J. Crohns Colitis 8, 1464–1470. 10.1016/j.crohns.2014.05.010 24958064

[B162] Uhr J. W. Dancis J. Franklin E. C. Finkelstein M. S. Lewis E. W. (1962). The antibody response to bacteriophage phi-X 174 in newborn premature infants. J. Clin. Invest. 41, 1509–1513. 10.1172/JCI104606 13923602 PMC291062

[B163] Underhill D. M. Iliev I. D. (2014). The mycobiota: interactions between commensal fungi and the host immune system. Nat. Rev. Immunol. 14, 405–416. 10.1038/nri3684 24854590 PMC4332855

[B164] Varela E. Manichanh C. Gallart M. Torrejón A. Borruel N. Casellas F.. (2013). Colonisation by *Faecalibacterium prausnitzii* and maintenance of clinical remission in patients with ulcerative colitis. Aliment. Pharmacol. Ther. 38, 151–161. 10.1111/apt.12365 23725320

[B165] Velthuis J. H. Unger W. W. Abreu J. R. Duinkerken G. Franken K. Peakman M. (2010). Simultaneous detection of circulating autoreactive CD8(+) T-cells specific for different islet cell-associated epitopes using combinatorial MHC multimers. Diabetes 59, 1721–1730. 10.2337/db09-1486 20357361 PMC2889772

[B166] Virgin H. W. (2014). The virome in mammalian physiology and disease. Cell 157, 142–150. 10.1016/j.cell.2014.02.032 24679532 PMC3977141

[B167] Vrieze A. Van Nood E. Holleman F. (2013). Transfer of intestinal microbiota from lean donors increases insulin sensitivity in individuals with metabolic syndrome (vol 143, pg 913, 2012). Gastroenterology 144, 250–250. 10.1053/j.gastro.2012.11.032 22728514

[B168] Wagner J. Maksimovic J. Farries G. Sim W. H. Bishop R. F. Cameron D. J.. (2013). Bacteriophages in gut samples from pediatric Crohn's disease patients: metagenomic analysis using 454 pyrosequencing. Inflamm. Bowel Dis. 19, 1598–1608. 10.1097/MIB.0b013e318292477c 23749273

[B169] Waller A. S. Yamada T. Kristensen D. M. Kultima J. R. Sunagawa S. Koonin E. V.. (2014). Classification and quantification of bacteriophage taxa in human gut metagenomes. ISME J. 8, 1391–1402. 10.1038/ismej.2014.30 24621522 PMC4069399

[B170] Wehkamp J. Harder J. Wehkamp K. B. Wehkamp-von Meissner B. Schlee M. Enders C.. (2004). NF-κB-and AP-1-mediated induction of human beta defensin-2 in intestinal epithelial cells by *Escherichia coli* Nissle 1917: a novel effect of a probiotic bacterium. Infect. Immun. 72, 5750–5758. 10.1128/IAI.72.10.5750-5758.2004 15385474 PMC517557

[B171] Weinstock J. V. Elliott D. E. (2009). Helminths and the IBD hygiene hypothesis. Inflamm. Bowel Dis. 15, 128–133. 10.1002/ibd.20633 18680198

[B172] Weinstock J. V. Summers R. W. Elliott D. E. Qadir K. Urban J. F. Thompson R. (2002). The possible link between de-worming and the emergence of immunological disease. J. Lab. Clin. Med. 139, 334–338. 10.1067/mlc.2002.124343 12066130

[B173] Willing B. Halfvarson J. Dicksved J. Rosenquist M. Järnerot G. Engstrand L.. (2009). Twin studies reveal specific imbalances in the mucosa-associated microbiota of patients with ileal Crohn's disease. Inflamm. Bowel Dis. 15, 653–660. 10.1002/ibd.20783 19023901

[B174] Wu G. D. Chen J. Hoffmann C. Bittinger K. Chen Y. Y. Keilbaugh S. A.. (2011). Linking long-term dietary patterns with gut microbial enterotypes. Science 334, 105–108. 10.1126/science.1208344 21885731 PMC3368382

[B175] Xenoulis P. G. Palculict B. Allenspach K. Steiner J. M. Van House A. M. Suchodolski J. S. (2008). Molecular-phylogenetic characterization of microbial communities imbalances in the small intestine of dogs with inflammatory bowel disease. FEMS Microbiol. Ecol. 66, 579–589. 10.1111/j.1574-6941.2008.00556.x 18647355

[B176] Yan X. Sivignon A. Yamakawa N. Crepet A. Travelet C. Borsali R.. (2015). Glycopolymers as antiadhesives of *E. coli* strains inducing inflammatory bowel diseases. Biomacromolecules 16, 1827–1836. 10.1021/acs.biomac.5b00413 25961760

[B177] Yang X. D. Yang Y. P. Wang Y. Y. Zhan B. Gu Y. Cheng Y. L.. (2014)Excretory/secretory products from trichinella spiralis adult worms ameliorate DSS-induced colitis in mice. PLoS ONE 9:96454. 10.1371/journal.pone.0096454 24788117 PMC4008629

[B178] Yang YJ. Kim M.-S. Kim E. Cheon J. H. Lee Y.-S. Choi S. S.. (2016). Enteric viruses ameliorate gut inflammation via toll-like receptor 3 and toll-like receptor 7-mediated interferon-β production. Immunity 44, 889–900. 10.1016/j.immuni.2016.03.009 27084119

[B179] Yatsunenko T. Rey F. E. Manary M. J. Trehan I. Dominguez-Bello M. G. Contreras M.. (2012). Human gut microbiome viewed across age and geography. Nature 486, 222–227. 10.1038/nature11053 22699611 PMC3376388

[B180] Yu T. Guo F. Yu Y. Sun T. Ma D. Han J.. (2017). *Fusobacterium* nucleatum promotes chemoresistance to colorectal cancer by modulating autophagy. Cell 170, 548–563.e16. 10.1016/j.cell.2017.07.008 28753429 PMC5767127

[B181] Zhang Y. F. LeJeune J. T. (2008). Transduction of bla(CMY-2), tet(A), and tet(B) from Salmonella enterica subspecies enterica serovar Heidelberg to S-Typhimurium. Vet. Microbiol. 129, 418–425. 10.1016/j.vetmic.2007.11.032 18187273

[B182] Zhu W. H. Winter M. G. Byndloss M. X. Spiga L. Duerkop B. A. Hughes E. R.. (2018). Precision editing of the gut microbiota ameliorates colitis. Nature 553, 208–211. 10.1038/nature25172 29323293 PMC5804340

[B183] Zuo T. Kamm M. A. Colombel J.-F. Ng S. C. (2018). Urbanization and the gut microbiota in health and inflammatory bowel disease. Nat. Rev. Gastro. Hepat. 15:440–452. 10.1038/s41575-018-0003-z 29670252

[B184] Zuo T. Wong S. H. Lam L. Y. K. Lui R. Cheung K. Tang W. (2017). Bacteriophage transfer during fecal microbiota transplantation is associated with treatment response in clostridium difficile infection. Gastroenterology 152, S140–S141. 10.1016/S0016-5085(17)30798-9 PMC5868238 28539351

